# Re-prioritizing traffic stops to reduce motor vehicle crash outcomes and racial disparities

**DOI:** 10.1186/s40621-019-0227-6

**Published:** 2020-01-20

**Authors:** Mike Dolan Fliss, Frank Baumgartner, Paul Delamater, Steve Marshall, Charles Poole, Whitney Robinson

**Affiliations:** 1 Injury Prevention Research Center, University of North Carolina, Chapel Hill, 521 S Greensboro St, Carrboro, NC 27510 USA; 2 Department of Political Science, University of North Carolina, Chapel Hill, 235 E Cameron Ave, Chapel Hill, NC 27514 USA; 3 Department of Geography, University of North Carolina, Chapel Hill, Carolina Hall, CB# 3220, Chapel Hill, NC 27599 USA; 4 Carolina Population Center, University of North Carolina, Chapel Hill, 123 W Franklin St, Chapel Hill, NC 27516 USA; 5 Department of Epidemiology, University of North Carolina, Chapel Hill, 170 Rosenau Hall, CB #7400 | 135 Dauer Drive, Chapel Hill, NC 27599 USA

**Keywords:** Traffic stop, Motor vehicle crash, Disparity, Crime, Synthetic control, Law enforcement, Policing, Race, Public health critical race praxis

## Abstract

**Background:**

Law enforcement traffic stops are one of the most common entryways to the US justice system. Conventional frameworks suggest traffic stops promote public safety by reducing dangerous driving practices and non-vehicular crime. Law enforcement agencies have wide latitude in enforcement, including prioritization of stop types: (1) safety (e.g. moving violation) stops, (2) investigatory stops, or (3) economic (regulatory and equipment) stops. In order to prevent traffic crash fatalities and reduce racial disparities, the police department of Fayetteville, North Carolina significantly re-prioritized safety stops.

**Methods:**

Annual traffic stop, motor vehicle crash, and crime data from 2002 to 2016 were combined to examine intervention (2013–2016) effects. Fayetteville was compared against synthetic control agencies built from 8 similar North Carolina agencies by weighted matching on pre-intervention period trends and comparison against post-intervention trends.

**Results:**

On average over the intervention period as compared to synthetic controls, Fayetteville increased both the number of safety stops + 121% (95% confidence interval + 17%, + 318%) and the relative proportion of safety stops (+ 47%). Traffic crash and injury outcomes were reduced, including traffic fatalities − 28% (− 64%, + 43%), injurious crashes − 23% (− 49%, + 16%), and total crashes − 13% (− 48%, + 21%). Disparity measures were reduced, including Black percent of traffic stops − 7% (− 9%, − 5%) and Black vs. White traffic stop rate ratio − 21% (− 29%, − 13%). In contrast to the Ferguson Effect hypothesis, the relative de-prioritization of investigatory stops was not associated with an increase in non-traffic crime outcomes, which were reduced or unchanged, including index crimes − 10% (− 25%, + 8%) and violent crimes − 2% (− 33%, + 43%). Confidence intervals were estimated using a different technique and, given small samples, may be asymmetrical.

**Conclusions:**

The re-prioritization of traffic stop types by law enforcement agencies may have positive public health consequences both for motor vehicle injury and racial disparity outcomes while having little impact on non-traffic crime.

## Background

Law enforcement traffic stops are one of the most common first contacts with the US justice system (Davis 2018). Community-led movements (American Civil Liberties Union of Illinois 2014), national press (LaFraniere and Lehren 2015), peer-reviewed research (Baumgartner et al. 2018a, 2018b) and the Department of Justice (US Department of Justice, Civil Rights Division 2015) have all suggested that traffic stops are most burdensome to low-income and racial-ethnic minority drivers and their communities. In this paper we provide a brief background on law enforcement traffic stops through conventional and critical public health lens and evaluate an intervention designed to reduce racial-ethnic disparities in traffic stops while reducing traffic crash injury outcomes.

Conventional frameworks suggest traffic stops promote public safety by reducing dangerous driving practices and non-vehicular crimes. Assumptions of criminal justice deterrence theory (Becker 1968) underlie these conventional frameworks, treating dangerous driving and non-vehicular crimes as events where each actor rationally weighs the certainty of being caught, the celerity (speed) of that consequence, and the severity of punishment against the immediate positive consequences of their action. This conventional framework implies an objective world of traffic stop rationale where some have chosen to break the law, others have not, and traffic stops of all kinds have a wholly positive effect on public safety. These frameworks either ignore traffic stop disparities entirely or justify them as negative collateral consequences to otherwise legal and rational public safety interventions. In either case, conventional frameworks suggest these disparities merit little attention and action under an objective enforcement of the law.

### Law enforcement discretion, priorities, and disparities

In contrast to these conventional frameworks, public health authorities have called for analyses that center disparities and for engagement in anti-racist action (Jones 2001). The American Public Health Association (APHA) recently launched a National Campaign Against Racism (Jones 2018) that suggests going beyond an individual focus (e.g. who is or isn’t racist) to ask, “how is racism operating here?” within structures, policies, practices, norms and values (Jones 2018). One mechanism for how racism operates in the application of justice is through individual and agency discretion. In contrast to conventional objective frameworks, law enforcement agencies have wide, subjective discretion in the selective enforcement of traffic stops. Supreme court cases in 1968 and 1996 (Chief Justice Warren 1968; Justice Scalia 1996) enabled US law enforcement, under any reasonable suspicion and the loosest definitions of crime profiles, to escalate any minor traffic violation into a traffic stop (Meares 2014). Nearly all driving trips include actions interpretable as infractions, whether small wavering within lanes or movement over or under posted speed limits (Baumgartner et al. 2018a, 2018b; Meares 2014). Taken together, these rulings legally permit law enforcement nearly complete discretion over traffic stop enforcement, even if the public views those stops as unfair (Meares et al. 2015).

These enforcement and patrol priorities differentially expose populations to different patrol densities and thresholds of interaction based on neighborhood-level factors. Neighborhood-level segregation by race-ethnicity and income, coupled with institutional policies prioritizing certain spaces and incident types, operates alongside any additional disparities caused by interpersonal bias based on perceived race-ethnicity phenotypes. Indeed, previous studies have quantitatively refuted the idea that individual outlier officers (e.g. the “bad apple” hypothesis) sufficiently explain the large racial-ethnic disparities found in traffic stops (Baumgartner et al. 2018a, 2018b). Still, all individual officers exercise subjective discretion in their traffic stop enforcement, and all do so partly informed by their race-ethnicity, gender, and socio-economic position personal biases, both implicit and explicit (Schafer et al. 2006). In addition, individual officers do not operate within a vacuum. Officers operate within, or at least influenced by, the implicit norms and explicit policies of their agencies (Schafer et al. 2006). Those formal and informal policies include neighborhood-specific patrol deployments and the relative emphasis of public safety and control priorities.

The Public Health Critical Race Praxis (PHCRP), based on Critical Race Theory (Richard Delgado 2016) provides a standardized framework to investigate these traffic stop dynamics (Ford and Airhihenbuwa 2018, 2010) and critique conventional frameworks (Muhammad et al. 2018). PHCRP principles explicitly acknowledge the social construction of knowledge, structural determinism, critical analysis, and disciplinary self-critique (Ford and Airhihenbuwa 2010). In keeping with these principles, and in contrast to conventional frameworks, we recognize law enforcement agency priorities and exercise of discretion are constructed over time, malleable in the present and future, influence officers and communities beyond individual interactions, and deserving of critical analysis.

Considering the relative and absolute frequency of traffic stops by the type of stop is one method of understanding an agency’s implicit and explicit priorities. For the purpose of this discussion, we divide traffic stops into three categories. (1) “Safety stops” include violations of speed limits, stop lights, driving while impaired, and safe movement. (2) “Investigatory stops” include explicit investigation, unspecified rationales, and discretionary seatbelt enforcement, since in prior studies seatbelt stops are most similar to investigatory stops in their disparate application (Baumgartner et al. 2018a, 2018b) and may have mixed evidence as primary stop rationale (Harper 2019). Lastly, (3) “economic stops” are disproportionately consequences of economic circumstances, including not carrying insurance, expired motor vehicle registrations, or equipment malfunctions. Under conventional frameworks these three stop types may be associated with public safety injury and crime outcomes. For instance, safety stops ostensibly reduce motor vehicle and pedestrian crashes. Similarly, investigatory stops may be designed to reduce non-traffic crime, discover and detain individuals after having committed certain crimes, or reduce traffic injury severity by increasing seatbelt use. Finally, economic stops could be framed conventionally as reducing traffic crashes because of equipment failures. Because of their link to public safety outcomes, the relative and absolute frequency of these traffic stop types represent a set of often implicit public health prioritizations.

However, disparities in traffic stops may also differ by these stop types: For instance, Black and Hispanic drivers constitute a larger proportion of investigatory and economic stops than safety related stops in the North Carolina, and are disproportionately over-represented in all stop types relative to the North Carolina population (Baumgartner et al. 2018a, 2018b). In contrast with conventional frameworks that conceive economic stops as protective and unbiased, critical intersectional frameworks acknowledge the link between race-ethnicity and income disparities. Since Black and Hispanic individuals are often disproportionally represented in low-income and low-wealth populations, they may also be disproportionally at risk of economic stops. Due to segregation, they may be more likely to live in lower-resourced areas where investigatory stops are more prevalent, creating multi-level disparity dynamics. These higher-disparity stops are not infrequent: statewide, previous analysis of the North Carolina traffic stop dataset statewide (Baumgartner et al. 2018a, 2018b) demonstrates that economic and investigatory stops make up nearly half of all traffic stops. These disparities by traffic stop type suggest that an agency’s relative traffic stop type priorities, whether implicit or explicit, represent not only prioritizations of public safety outcomes but also potentially disparate population targeting.

When agency and officer enforcement priorities differ from community priorities, this violates principles of community self-determination and consequently threatens community trust and perceived legitimacy of law enforcement (Fontaine et al. 2017; Hamm et al. 2017). Trust may also be challenged within agencies, such as when new agency priorities differ from individual officer priorities (Kramer 1999). Law enforcement agencies or individual officers may respond to community mistrust and calls for increased community accountability by scaling back their public safety services (such as certain traffic stops) believed to be essential for violent crime control. This dynamic, named the Ferguson Effect (Gross and Mann 2017), is therefore observable (and testable) in two parts: after increased public scrutiny or reprioritization of public safety activities, there will be a (1) drop in law enforcement activities and (2) an increase in the negative outcomes (e.g. violent crime) those activities were meant to protect against. Studies have shown evidence of Ferguson Effects in the attitudes and actions of officers (drops in productivity, reduced motivation, belief crime will rise as officers “de-police”), though this effect was moderated by their belief in whether communities afford legitimacy to policing (Nix and Wolfe 2018). In contrast, the evidence for increases in negative crime outcomes after de-policing is mixed, confounded by income inequality and racial segregation (Gross and Mann 2017). A recent Missouri study found no effect on crime outcomes at all when traffic stops, searches, and arrests are reduced specifically (Shjarback et al. 2017). Because the intervention considered just such a reprioritization within an agency after community members challenged police legitimacy, we acknowledge this Ferguson Effect as a relevant dynamic for consideration and evaluate it as a secondary aim.

### Fayetteville intervention

Given finite budget and staffing realities, law enforcement administrators may choose to direct agency traffic stop programs to target certain public safety outcomes by prioritizing traffic stops by type or directing patrol patterns to maximize traffic stop efficiency. In keeping with this opportunity, city leaders in Fayetteville, North Carolina were called to respond to the city’s consistently high motor vehicle crash rate (Barksdale 2013). Simultaneously, tensions between community groups and police came to a head as city council intervened to halt searches that disproportionately targeted Black residents. Soon after, the police chief and second-in-command stepped down (Top two Fayetteville police officials leave amid controversy n.d.).

After newly being appointed in 2013 and faced with issues of motor vehicle crashes and eroded community trust, Chief Harold Medlock voluntarily requested a review of his department practices and policies by the US Department of Justice Office of Community Oriented Policing Services’ (COPS Office) (COPS Office: Ethics and Integrity Training 2019) Collaborative Reform Initiative for Technical Assistance (CRI-TA) (Rodriguez et al. 2015). That report provided preliminary evidence of racial disparities in traffic stops compared to Fayetteville’s residential data, though also documented the beginnings of a reduction starting with his tenure in 2013. The report also documented that Fayetteville began to require officers collect Global Positioning System (GPS) data on all traffic stops, an element still not required on the state form; this is corroborated in Fayetteville’s written policies for traffic stops, where failure to record this data are grounds for negative performance review (Fayetteville Police Department Administrative Bureau 2015). Those data could then be used alongside its Crash Analysis Reduction Strategy (CARS) program, where ten intersections with the most crashes were used for targeted traffic stop enforcement each week (Fayetteville Police Department 2019). To increase transparency and accountability, press releases were disseminated each week detailing these locations, with three intersections targeted each day. The press releases also detailed the written warnings and state citations issued the prior week.

Because of Chief Medlock’s focus on traffic crash reductions and improving community trust exacerbated by racial disparities in traffic stops and other outcomes, he gave guidance to highly prioritize safety stops in order to prevent traffic crash fatalities and reduce racial disparities during his tenure from 2013 to 2016 (Barksdale 2016). We hereafter refer to this collection of changes to agency traffic stop activities, associated policies, workflows, staffing changes, and required organizational change work as the Fayetteville intervention. Notably this intervention included mechanisms we are not measuring in this analysis, including both quantifiable changes (e.g. increased spatial clustering of safety traffic stops around high crash locations) and changes more difficult to quantify (e.g. changing internal organization culture and norms). Therefore, though we track four quantitative measures describing their traffic stop prioritization profile to gauge intervention implementation over the study period, they are best seen as representative indicators of the intervention, not the full substance or mechanism of the intervention.

The purpose of this paper was to evaluate this Fayetteville intervention alongside a broader examination of the relationship of law enforcement traffic stops and public health outcomes.

## Methods

The intervention impact was assessed by comparing traffic stop, motor vehicle crash, and crime measures from Fayetteville Police Department to a composite control agency built by a weighted combination of data from eight similarly large North Carolina police departments that did not enact Fayetteville’s reprioritization intervention.

Four domain areas were chosen to assess the intervention’s impact. Traffic stop prioritization profile measures were chosen to provide evidence the intervention was not only designed and publicized but implemented. Traffic stop disparity measures were chosen to assess questions of improved equity. Motor vehicle crash measures were chosen to assess crashes averted and lives saved. Crime measures were chosen in order to explore the possibility of a Ferguson Effect, the possibility that a de-prioritization of investigatory and economic stops was associated with an increase in crime.

Thirteen measures were chosen from those four domain areas to assess these questions in more detail. Traffic stop prioritization profile measures included (1) number of safety-related traffic stops, (2) percent of safety-related stops, (3) percent of regulatory and equipment stops, and (4) percent of investigatory stops. Measures of traffic stop disparities included (5) percent Black non-Hispanic stops and (6) the traffic stop rate ratio (TSRR) of Black non-Hispanic to White non-Hispanic stops. Motor vehicle crash measures included (7) total crashes, (8) crashes with injuries, and (9) crash-related fatalities. Lastly, crime-related measures included violent crime (10) counts and (11) rates and index crime (12) counts and (13) rates. Notably, Black non-Hispanic traffic stop disparities against White non-Hispanic referent, though only one of a number of useful disparities to consider by race, ethnicity, gender, and age (Baumgartner et al. 2018a, 2018b), were chosen because of previously documented disparities, the specific history of anti-Black racism in the United States, and the explicit focus in Fayetteville around those disparities.

When considering causal questions involving race-ethnicity, individual race-ethnicity comes to simultaneously represent a range of interrelated, but separate constructs (e.g. phenotype, self-identified race, socially assigned race, experiences of discrimination, structural racism, historical trauma, etc.) that have unique causal relationships (VanderWeele and Robinson 2014). We acknowledge this, but do not in this study divide the construct into its many components or bring in accessory datasets to improve its contextualization and construct precision.

### Data sources

Traffic stop data were obtained from the North Carolina State Bureau of Investigation (SBI) database, including over 20 million police traffic stops from 2002 to 2018, representing 308 of the 518 state, county, municipal, campus, and place-specific (e.g. state fairgrounds, capital building) police departments (NC State Bureau of Investigation 2019). By 2002, reporting was mandated by most North Carolina agencies, including all sheriff departments, state agencies, and municipal agencies with jurisdictions above 10,000 population, making it one of the oldest and most complete traffic stop databases in the nation (Baumgartner et al. 2018a, 2018b). Though it does not include all agencies, it represents the policing jurisdictions of 99% of the state population, excluding only the smallest cities and place-specific agencies. All traffic stop measures were derived solely or in part from this dataset.

One evaluation measure, the rate ratio of Black non-Hispanic vs. White non-Hispanic driver traffic stops, required accessory datasets to calculate. Per previous literature (Fliss 2019; US Department of Justice, Civil Rights Division 2015; Withrow and Williams 2015), commonly used, residential-based rates for traffic stops are fundamentally flawed since traffic stops are inherently tied to travel patterns. A supplemental dataset, the 2017 National Household Travel Survey, was used previously to produce NC-specific estimates of vehicle access and vehicle miles traveled by race-ethnicity group (Fliss 2019). Since NC elected to additionally fund the survey as an add-on partner for supplemental sampling (Dai and Roth 2017), survey results could be made representative of the state by multiplying by the pre-calculated weight factors specific to households, people, or trips to account for nuanced sampling strategies and non-response adjustments. Statewide estimates of vehicle access and total annual VMT (see Additional file 2: Table S2) were used as an adjustment factor to city- and year-specific residential data to derive city-year-specific estimates of drivers and total VMT by race-ethnicity (Fliss 2019). Specifically, 64.2% of Black non-Hispanic residents of Fayetteville were estimated to have access to a vehicle, contributing approximately 9775 VMT per year per driver on average. These driving adjustment factors were 82.2% and 10,819 VMT for White non-Hispanics, respectively.

Population demographic data for race-ethnicity-specific rate calculations were obtained from the United States American Communities Survey (ACS) and United States census, interpolating years 2002 to 2009 using 2000 and 2010 census data when ACS estimates were unavailable. Data on North Carolina motor vehicle crashes since 2002 were obtained from the University of North Carolina Highway Safety Research Center (HSRC) (UNC Highway Safety Research Center n.d.), and data on North Carolina crime counts and rates since 2002 were also obtained from the North Carolina SBI (NC State Bureau of Investigation 2019).

### Synthetic control

Authors have recently advocated for synthetic control’s utility to epidemiology (Rehkopf and Basu 2018) and it has been used specifically in assessing policy effects in both justice (Gius 2019; Muhammad et al. 2018) and public health (Abadie et al. 2010) contexts. In contrast to difference-in-difference (DiD) modeling, which can be conceived of a special case of synthetic control (Xu 2017), the synthetic control techniques compare measures from one or more intervention units over time (in this case, Fayetteville Police Department is the single unit) against measures derived from the weighted combination of 1 or more units from a pool of control units (Abadie et al. 2010). Synthetic control therefore has benefits over DiD in maximizing similarity to controls, loosening the parallel trends assumption, and a statistical basis for control selection (Robbins and Davenport 2018).

In this study, Fayetteville Police Department was the single intervention unit and eight similarly large cities in North Carolina served as the pool of potential controls (see Table 1). In this case and with small intervention (*N* = 1) and potential control pool numbers, the synthetic control technique finds 1 or more control agencies that, in linear weighted combination, generate a synthetic agency for each outcome measure with a pre-intervention trend that maximizes similarity against the intervention agency (or units, in larger studies) on for each measure. These same linear combinations of agency weights, determined by maximizing the pre-intervention period (2002–2012) matching, are then applied to the same agencies in the post-intervention period (2013–2016). The intervention agency can then be compared to the synthetic control agencies for each measure to generate an estimator of the difference between the Fayetteville with the intervention applied and a counterfactual Fayetteville as if it did not receive the intervention. Synthetic control methods, as a method of weighted matching, have the benefit of controlling for some unmeasured confounders (Abadie et al. 2010; Gius 2019) and can optionally be matched on one or more known time-varying or time-unvarying confounders besides pre-intervention outcome measures, though this was not done here for reasons described in the Discussion section. See Table 1 for the list of cities and summary measures from the pre-intervention period.

**Table 1:** Fayetteville and control agency demographics, traffic stops, crashes, and crime

	Demographic Measures			Traffic Stop Measures				Crash Measures			Crime Measures			
	Population	% Black	Median household income	Average annual safety stops	Safety stops (%)	Black driver stops (%)	Traffic stop rate ratio^a^	All crashes	Crashes with injuries	Fatalities from crashes	Index crimes	Index crime rate	Violent crime count	Violent crime rate
Intervention City														
Fayetteville	203,670	41%	$43,882	13,968	43.8	56.8	2.5	5298	1886	62	13,367	7848.1	1224	730.5
Control Cities														
Cary	155,822	8%	$94,617	9179	56.5	18.3	3.8	2355	615	9	2145	1663.8	115	88.9
Charlotte	808,834	35%	$55,599	47,177	43.4	50.4	2.7	22,943	8241	168	45,840	6219.8	6243	845.2
Durham	251,761	39%	$52,115	9329	48.7	57.0	2.8	7284	1979	38	13,233	6121.4	1758	806.2
Greensboro	282,177	41%	$42,802	21,043	55.6	50.9	2.1	7374	2930	53	14,873	5976.1	1767	708.4
High Point	108,982	33%	$43,322	9919	47.9	40.8	1.9	2327	908	23	5719	5805.5	653	659.8
Raleigh	441,326	28%	$58,641	26,374	44.6	45.0	2.9	13,675	3608	80	14,687	4063.9	1914	530.8
Wilmington	113,724	18%	$43,855	6674	52.6	25.7	1.9	3454	1298	32	6679	6707.7	774	773.5
Winston-Salem	238,474	34%	$40,898	13,616	46.1	45.0	2.1	5811	1798	42	15,026	7004.1	1690	786.6

^a^Traffic stop rate ratio is White non-Hispanic to Black non-Hispanic drivers adjusted to travel denominators instead of residential denominators. Average annual data from pre-intervention period (2002–2012). Abbreviations: *MHHI* Median household income

In this case, the synthetic control method was chosen to control for known global time trends (e.g. statewide changes in driving frequency) that a single-unit difference-in-difference analysis would have left uncontrolled for. As example, driving frequency may have changed statewide, or at least in multiple cities in this analysis, over the intervention period as a function of changes in employment due to the recession and its recovery. Comparing Fayetteville’s pre-intervention trend to only its own post-intervention trend would erroneously conflate any reduction in crashes of Fayetteville’s intervention to the reduction in crashes due to global changes in statewide driving. Synthetic control provides some control of this kind of confounding. Because the specific causal relationships of the intervention and its covariates are largely unmapped and because of the relatively small number of observations (acknowledging an intervention *n* = 1), no attempt was made to control for other specific time-varying or time-unvarying confounders between agencies beyond the confounding control that weighted matching on pre-intervention period provides for these global and potentially time-varying confounders. Independent synthetic control agencies were created for each measure for this same reason; simultaneous matching against all measures implies shared confounders between them, which was not known (and was not expected by authors) to be the case.

The post-intervention synthetic control annual average, annual difference between intervention and control, percent change with confidence interval, placebo test permutation *p*-value (calculated by assigning intervention status to each control agency and recalculating the post-intervention model), and linear trend *p*-value were calculated for each reprioritization, crash, disparity, and crime measure. 95% confidence intervals were estimated using Taylor series linearization as having relatively few units limit resampling- and placebo permutation-based methods. Given the number of units, these point estimates may not exactly match those derived from the synthetic control weighting-based method and therefore confidence intervals may be unsymmetrical. The statistical package R (R Core Team 2018) and key libraries (Pebesma 2018; Robbins and Davenport 2018; Wickham 2017) were used for analysis.

## Results

Synthetic control generated measure-specific weight vectors using between 1 and 5 control agencies (see Additional file 1: Table S1), with the model average of 3.0 agencies. Table 2 presents annual averages, differences, and percent change comparing post-intervention Fayetteville to the post-intervention control agency for thirteen intervention-related measures. At the end of the intervention period over 80% of Fayetteville’s traffic stops were safety stops, up from a low of 30% in 2010. The Fayetteville intervention was associated with a 47% average increase in the proportion of safety stops and a striking 121.3% (17.3%, 318.1%) average increase in the number of safety stops. From a low of just over 9000 safety stops in 2006, at the end of the intervention period Fayetteville completed nearly 60,000 safety stops in 2016.

**Table 2:** Treatment vs. synthetic control: stop profile, crash outcome, and crime outcomes

	Fayetteville Police Department		Synthetic Control	Difference between Fayetteville and Synthetic Control				
	Pre-intervention annual average	Post-intervention annual average	Post-intervention annual average	Annual Difference	Percent Change and 95% CI (%)		Linear test *p*-value	Permutation test *p*-value
Traffic Stop Profile								
Total Safety Stops	13,968 (100%)	34,930 (100%)	15,786 (100%)	+ 19,144	+ 121.3	(+ 17.1, + 318.1)	0.0055	< 0.0001
% Safety Stops	6119 (43.8%)	23,786 (68.1%)	7296 (46.2%)	+ 21.9%	+ 47.3	(+ 20.0, + 80.9)	0.0001	< 0.0001
% Regulatory & Equip. Stops	6073 (43.5%)	9583 (27.4%)	6951 (44%)	−16.6%	−37.7	(−54.6, − 14.5)	0.0012	< 0.0001
% Discretionary	1776 (12.7%)	1562 (4.5%)	1367 (8.7%)	−4.2%	−48.4	(−55.5, − 40.1)	< 0.0001	< 0.0001
Measures of Traffic Stop Disparity								
% Black non-Hispanic Stops	56.8%	54.7%	58.8%	−4.1%	−7.0	(−8.9, −5.0)	< 0.0001	0.250
Black non-Hispanic TSRR	2.5	2.2	2.8	n/a	−21.3	(−28.5, −13.3)	< 0.0001	0.125
Motor Vehicle Crash Outcomes								
Crashes (all)	5298 (100%)	5160 (100%)	5925 (100%)	−765.0	−12.9	(−37.5, + 21.3)	0.4439	0.125
Crashes (w/ injuries)	1886 (35.6%)	1639 (31.8%)	2118 (41%)	−479.3	−22.6	(−48.5, + 16.3)	0.2763	0.125
Traffic Fatalities	62.3	48.8	68.0	−19.3	−28.3	(−64.1, + 43.2)	0.4146	0.125
Crime Outcomes								
Violent Crimes	1223.6	1233.5	1257.3	−23.8	−1.9	(−32.8, + 43.2)	0.9218	> 0.99
Violent Crime Rate (per 1000)	730.5	596.9	582.4	+ 14.5	+ 2.5	(−14.0, + 22.2)	0.7815	0.750
Index Crimes	13,367.4	11,658.0	12,896.4	− 1238.4	−9.6	(−24.5, + 8.2)	0.2923	0.500
Index Crime Rate (per 1000)	7848.1	5637.3	5933.4	−296.1	−5.0	(−12.8, + 3.5)	0.2482	0.750

Table includes both annual averages pre-intervention (2002–2012) and post-intervention (2013–2016). Note: confidence intervals are not symmetrical around point estimates because different methods were used to produce each and small numbers further limited convergence

Both measures of Black non-Hispanic traffic stop disparities were reduced in Fayetteville as compared to the synthetic control agencies: the percent of traffic stops reduced 7.0% and the driving-adjusted traffic stop rate ratio was reduced 21%. Linearization estimates were similar and associated confidence intervals were relatively small.

All three measures of negative traffic outcomes were also reduced relative to synthetic controls: total crashes were reduced 13% (765 fewer each year), injurious crashes were reduced 23% (479 fewer each year), and traffic fatalities were reduced 28% (representing 19 fewer fatalities each year). The percent change in metrics associated with motor vehicle crashes were large but had wider confidence intervals and moderate agreement with Taylor linearization estimates.

Non-traffic crime outcomes showed little change. Index crime counts and rates were reduced 10% and 5% respectively, though confidence intervals were high. The Fayetteville violent crime count and rate were effectively indistinguishable from the control, with small estimates, wide relative confidence intervals, permutation test *p*-value > 0.99 and linear p-test of 0.96. Because of this, synthetic control estimates poorly matched the Taylor linearization estimates and small counts and rates disagreed in direction of association.

**Fig. 1:**
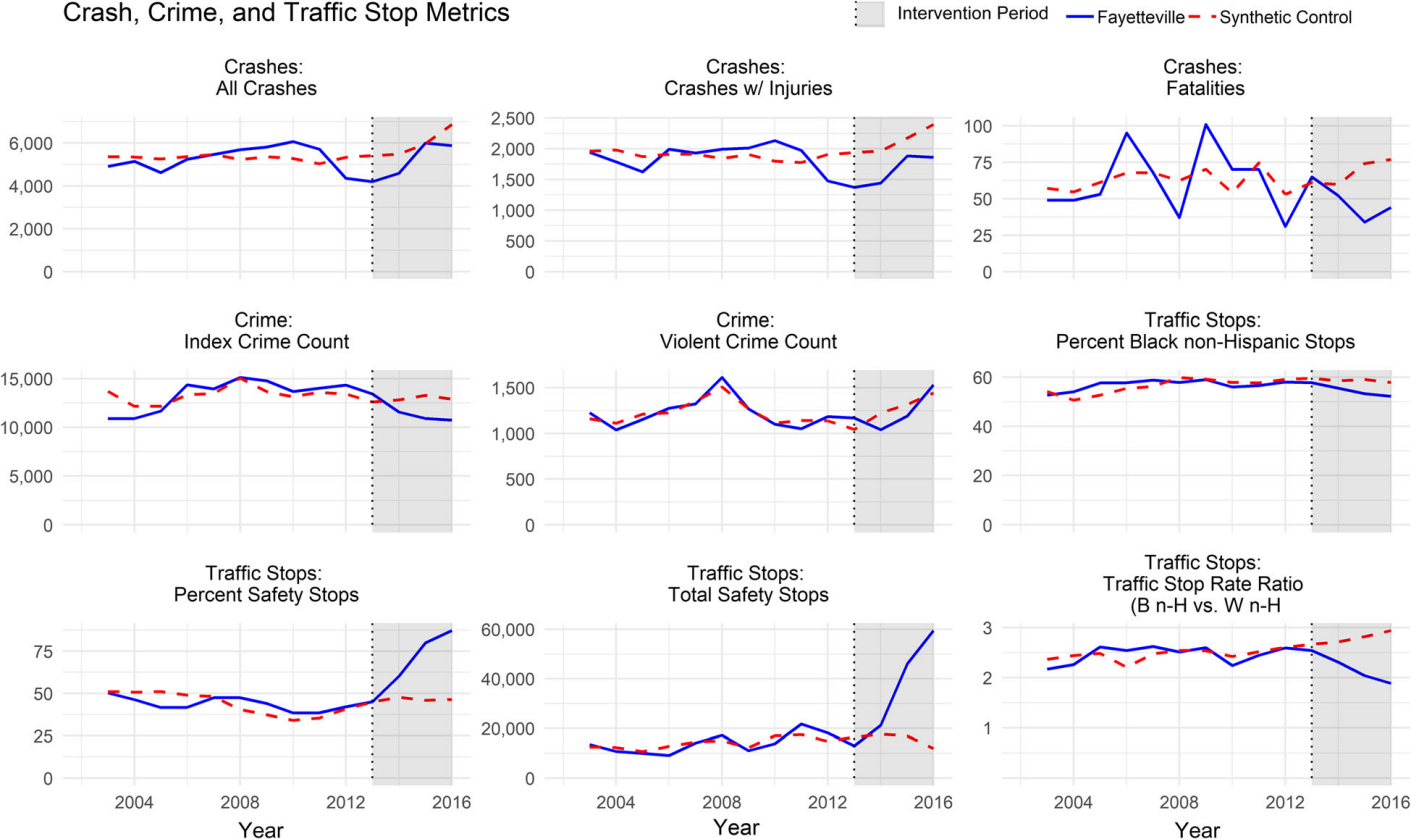
Crash, Crime, and Traffic Stop Metrics pre- and post-intervention period. Fayetteville Police Department is compared to a synthetic control department built by the 8 most similarly urban, high population, North Carolina police departments best matched for the specific metric during the pre-intervention period, from 2002-2012. Differences between the synthetic control (e.g. counter factual Fayetteville) and Fayetteville during the post-intervention period (i.e. 2013-2016) represent the modeled effect of the intervention

Figure 1 shows the trend of nine of these measures. The respective synthetic control agencies closely matched Fayetteville’s pre-intervention trends for most measures. Relatively small numbers of traffic fatalities among many agencies created more variation in the pre-intervention match for that measure. Divergence in the intervention period (in grey) demonstrates the intervention’s modeled effect.

**Fig. 2:**
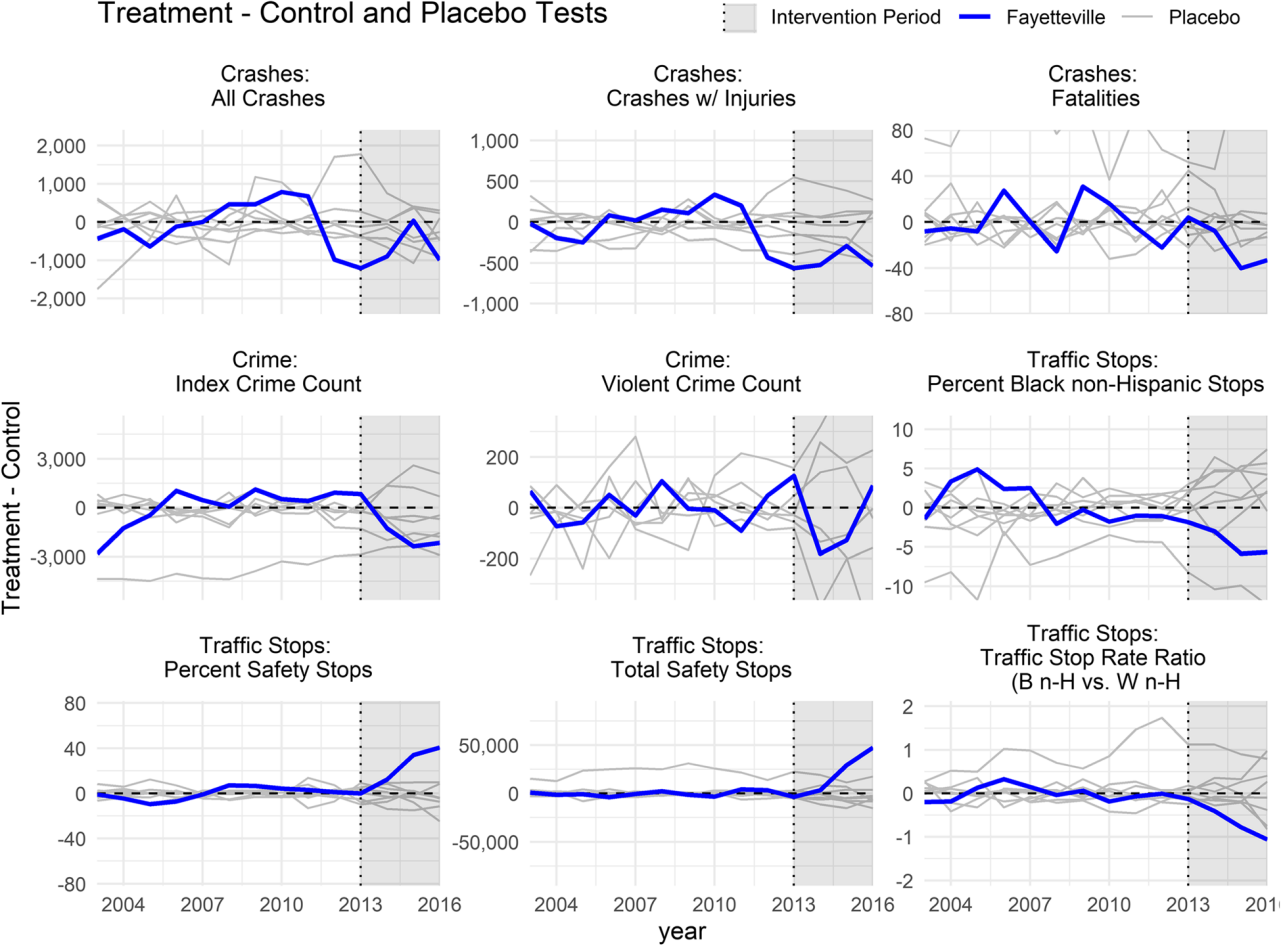
Treatment – Control Trends and Placebo Tests, pre- and post-intervention period. All permutations of non-intervention law enforcement agencies were treated as if they had enacted the intervention during the period, even though they hadn’t (placebo treatment), then likewise matched on pre-intervention period metrics using the same synthetic control process. The estimated change (treatment – control, ideally zero during the pre-intervention period) is graphed for both actually treated and placebo treatments. Some placebo comparisons produce outlier trend lines because the control pool was selected for Fayetteville, and placebos may be inadequately matched

Figure 2 shows the estimated effect (Treatment – control) for these same nine measures, as well as permutation tests of non-intervention agencies modeled under the same synthetic control framework with a placebo intervention. These placebo trends are graphical representations of the summary measure placebo tests presented in Table 2. Post-intervention clustering of the placebo trends, clustering of the pre-intervention trend around zero, and a sharp direction change of the intervention unit post-intervention represent stronger model fit.

## Discussion

Traffic stop profile measures confirmed the implementation of the intervention strategy. Both the relative percent of safety stops and the absolute number of safety stops completed marked increased in Fayetteville in comparison to the measure-specific synthetic control agencies. This increase in the percent of safety stops was matched with a corresponding relative reduction in economic and investigatory stops.

Motor vehicle crash outcomes were all reduced, though confidence intervals were relatively wider. Measures of traffic stop disparities were also reduced, suggesting a focus on safety stops (and relative de-prioritization of investigatory and economic stops) was a viable strategy to reduce Black non-Hispanic disparities in their traffic stop program.

Neither index crimes nor violent crimes changed appreciably during the intervention relative to the synthetic control agencies: three measure point estimates saw small reductions and one saw a small increase, but these nominal changes were much smaller than their associated confidence intervals. The disagreement in direction of the small change violent crime counts (decrease) and rates (increase) demonstrates that the measure was largely unchanged; small variation in population denominators explain the metric direction disagreement and the intervention effect on violent crime was effectively indistinguishable. This study does not provide any evidence of a negative effect on crime for de-prioritizing investigatory and economic stops. However, a more detailed view of the trend of the reduction in the total number of stops during the transition into the intervention suggests the first half of the Ferguson Effect, a reduction in output by some officers in response to community outcry and public attention, may have occurred in the first intervention year. Staffing changes as agency culture changed may also have occurred during the intervention roll-out period, producing or contributing to this reduction in output before the subsequent large increase in safety stops.

These results suggest redesigning a traffic stop program for public health impact may reduce negative motor vehicle crash outcomes, simultaneously reduce some negative consequences of traffic stop programs (e.g. race-ethnic disparities, reduced economic stop burden on communities), and the relative de-prioritization may not have an significant impact on crime rates. Safety traffic stops, especially when directed at high crash areas using regular review and traffic stop GPS data for evaluation, may be a more effective public safety tool than economic or investigatory stops. If investigatory stops can be de-prioritized with little impact on crime, but carry with them negative consequences to community trust, those traffic programs may be de-emphasized even without a relative prioritization of safety stops.

However, these apparent public health wins can be fleeting, as transitions in administrators may bring entirely new or adjusted priorities. Since Chief Medlock’s retirement in 2016, the percent of safety-related stops has dropped and the percent of Black drivers stopped has increased (Open Data Policing 2019). Future analyses may explore whether these new changes are associated with increases, decreases, or neither in crash, injury, and crime measures. Adherence to consistent public health priorities, especially when those relative priorities and implicit logics are made explicit, may help administrators transition while keeping interventions consistent.

### Negative consequences of traffic stops

This study posits a relationship between certain stop types and public health outcomes under a conventional framework. However, that conventional framework ignores or downplays the real, negative consequences of traffic stop enforcement in practice. Regulatory and equipment stops, and their associated fines, are a direct form of criminalizing individual and community economic poverty. Beyond the immediate impacts, the harm of economic stops creates a negative spiral operating within communities collectively and individuals specifically, extracting wealth and people’s bodies from low-income communities as the inability to pay mounting traffic tickets escalate into denied registration and warrants for arrest. The United State Justice Department Civil Rights Division cited this extreme and racialized extraction of wealth through traffic stops in its review of the Ferguson Police Department (US Department of Justice, Civil Rights Division 2015). When used unaccountably (e.g. recording no GPS data, as is the norm in NC), moving and safety violation stops can be enforced in an area with few motor vehicle crashes to justify them. Lastly, investigatory stops may have strikingly low contraband hit rates or racialized application (Baumgartner et al. 2018a, 2018b), which subject some to antagonistic law enforcement interactions over years (Peralta and Corley 2016) without contraband to show for the interaction.

Beyond the serious financial and carceral consequences, at their most severe, traffic stops can have fatal consequences for motorists, even when unarmed. Sandra Bland, an unarmed Black woman who died in jail after a routine traffic stop, had multiple other unpaid traffic tickets at the time of her arrest, including for operating a vehicle without a license and lack of insurance (Katy Smyser 2015). Walter Scott, an unarmed Black man, was shot to death, in the back, by a South Carolina police officer after a traffic stop for a non-functioning brake light (Blinder 2017). Philando Castile was pulled over forty times, for reasons including speeding, driving without a muffler and not wearing a seat belt, in the years running up to his fatal shooting during a traffic stop (Peralta and Corley 2016). An uncritical increase in traffic stop enforcement means increased interactions with law enforcement, creating more opportunities for escalated and fatal encounters that may disproportionately impact low-income people and people of color given structural disparities and both implicit and explicit bias. The associated loss of community trust has real public health consequences, including fewer calls for timely emergency services (Desmond et al. 2016). Beyond the negative consequences acknowledged to be more objective, public safety interventions driven by traffic stops should acknowledge the disparate, subjective, emotional experience drivers of color experience. Recent studies now document how these disparities in chronic stress get biologically embedded (i.e. “get under the skin”) and have measurable and negative consequences for individual health (Hertzman and Boyce 2010; Krieger et al. 2015; Nuru-Jeter et al. 2009), including specifically symptoms of post-traumatic stress disorder associated with increased interactions with police (Hirschtick et al. 2019).

### Program effectiveness, program efficiency

Central to this discussion are questions of absolute and relative intervention efficacy and efficiency. In Fayetteville’s case, their safety stop program was likely more efficient because of its use of crash data to inform prioritization of intersections and the geocoded stop data to ensure intervention fidelity. However, safety related traffic stops are not the only method to reduce motor vehicle crash injuries. The efficacy of even maximally efficient traffic stop programs must be weighed against strategies from other sectors such as public education campaigns and built environment investments, which may be either or both more efficacious and cost-efficient (Centers for Disease Control and Prevention, National Center for Injury Prevention and Control 2019). Likewise, focusing on policing interventions for public safety in the absence of infrastructure improvements, given historical (e.g. redlining) and present disparities in those investments raise equity concerns (Rothstein 2017).

When considering equitable investment in communities, this intervention to reprioritize traffic stops may best be a stop gap response to immediately reduce disparities and promote traffic crash outcomes but is not an ultimate solution. Though the intervention reduced racial disparities in Fayetteville compared by 21% of what they could have been, Black drivers still experienced over twice the incidence of traffic stops per vehicle miles traveled as White non-Hispanic drivers at the end of the study period. If not considering alternative interventions that may be more efficient, efficacious, or equitable, an investment in traffic stop programs in isolation may be capable of reducing motor vehicle crashes further but may require a totalitarian police state model stopping nearly all drivers for every possible infraction. Intervention considerations should include not only comparison of the positive efficacy and financial cost of programs but should weigh the negative collateral or intentional damages done. Traffic stop programs may be intentionally phased out or scaled back alongside infrastructure investments and other interventions that carry fewer negative and inequitable consequences to remain in alignment with public safety needs.

The same principles are true when considering other public safety outcomes: though policing has seen large funding increases and expanding scope of practice (Hinton 2016), policing should not be seen as either a panacea overall or the most efficacious intervention for non-vehicular crime and injury specifically. Police do not replace mental health workers, social workers, or public health workers capable of implementing evidence-based programs at the individual and community level for substance misuse and violence-related outcomes. As law enforcement agencies are increasingly accountable to the efficacies and efficiencies of their programs, it is in their best interest to focus on programs, including carefully-designed traffic stop programs, that have fewer negative consequences, more equitable outcomes, improved efficacy, and efficient implementation when compared to interventions from other sectors.

### Program priorities and the relative worth of life

In both law enforcement and public health, we implicitly and explicitly prioritize certain causes of disease, injury, and death over causes. Our prioritizations are revealed by our evidence and assumptions of efficacy and efficiency, by program funding and implementation, and ultimately by community investments enabled by political power. Even ignoring other sectors and intervention strategies besides traffic stops, police may compare the cost and efficacy of traffic stop programs in preventing injury and death by motor vehicle crash to preventing injury or death during a burglary, assault, homicide, or suicide. When considering who is targeted by interventions, public health recommends considering the burden of traffic stop preventable injuries, the exposure to traffic stops in the form of patrols patterns and priorities, and distributions of both exposure and outcome across population subgroups (Ward et al. 2019) alongside efficacy and cost. Because of unequal distribution of outcomes, exposure to interventions, differences in intervention effectiveness and efficiency, these priorities come to represent the relative value of lives by race-ethnicity and socio-economic position. As example, if community investment (including through law enforcement and traffic stop patrol programs) in preventing deaths by assault grossly outweighs investment in prevention of deaths by motor vehicle crashes, overdose, or heart disease, and especially when the underlying burden of assault injuries and mortality is comparably low, we implicitly prioritize the health and lives of populations seeking to prevent assault over other public health priorities and other populations.

These prioritization dynamics operate at multiple levels within and above agencies: within agencies as individual officer, patrol team, and precincts patterns; and above as clusters of agencies, statewide, nationwide, and between countries. At the national level we see these prioritizations in the focus on criminalizing drug use and addiction in urban, Black communities in the 1980s that lead to disproportionate incarceration of Black people at a level rarely seen anywhere else in the world (Hinton 2016). In contrast, the multiple phases of the opioid epidemic since 2000, hitting more (but not exclusively) rural and white communities, has been comparably treated as a public health crisis rather than a criminal justice one (Bailey et al. 2017; Netherland and Hansen 2017). Though this intervention analysis provided some contextual factors at the agency level, future research should not be limited to either implicit bias at the individual or policy effects at the agency level, but instead should continue to focus on questions or program priorities and implicit worth of human life at multiple and interacting levels.

Whether legally defensible or not, traffic stop programs may still be considered unjust and burdensome. They may ignore racial disparities in financial hardships, erode community trust, embody community stress, and trade injury and loss of life outcomes in some communities to promote or appear to promote the well-being of other communities. Even within the same community, for example, a seatbelt program that extracts large amounts of financial resources may cause serious harm to individual and community health and may outweigh the injury prevention benefit. Co-designing traffic stop programs along with impacted communities may alleviate some of these negative outcomes, though likely not all given the multiple underlying dynamics at play (Smith and Holmes 2014). It is precisely these implicit disparities in the value of people’s experiences, and ultimately their bodies and lives, that drives associated policy platforms calling for the end of criminalization and dehumanization of Black and low-income communities (The Movement for Black Lives 2019).

### Accountability

We argue that public health has a fundamental interest in detailed traffic stop data given associated public safety outcomes and equity considerations under both conventional and anti-racist frameworks (Ford and Airhihenbuwa 2010). However, not all states maintain active traffic stop databases like North Carolina’s. Further, most active traffic stop databases that do exist were started recently. When contrasted with many other public health surveillance systems, limited data on traffic stops suggest a relatively limited oversight of law enforcement activities. Public health has already acknowledged that data on deaths caused by officers are public health data that can and should be maintained (Feldman et al. 2019; Krieger et al. 2015), and that collecting law enforcement data in general is fundamental to accountability and trust (McGregor 2015). Data collection on traffic stops should also include some within-agency spatial component, as Fayetteville has elected to collect, such as spatial coordinates or an address or intersection that could be retroactively geocoded. Besides promoting accountability and transparency, such detailed data on traffic stop programs also benefits police agencies. Spatially-referenced traffic stop data can inform prediction and intervention models of public safety events like crashes and violent assaults and also ensure accountability within the agency and to community priorities. GPS tools for spatial referencing are increasingly low-cost, included in most cell phones, and retrospective geocoding are inexpensive. Recognizing the decreasing cost and increasing utility, the National Institute of Justice (NIJ) and the Bureau of Justice Assistance collaborated with the National Highway Traffic Safety Administration (NHTSA) to promote the Data-Driven Approaches to Crime and Traffic Safety (DDACTS) (Crime Mapping for DDACTS - Crime Mapping and Analysis NewsCrime Mapping and Analysis News n.d.) program. Agencies that capture detailed traffic stop data would be following these NIJ best practices.

As an example of the equity implications of public safety interventions, NHTSA put out a manual for state highway safety offices that included evidence of law enforcement traffic stop activities by types of traffic stop (Goodwin et al. 2015). This document informed updates of CDC guidelines around motor vehicle safety interventions (CDC Injury Center Motor Vehicle Safety 2019). Included as an evidence-based intervention are “a saturation patrol (also called a blanket patrol, ‘wolf pack,’ or dedicated DWI patrol)” (Goodwin et al. 2015). Likewise, movement from secondary to primary enforcement of seatbelt laws (e.g. allowing seatbelt ticketing when no other infraction is present) is associated with more seatbelt usage and reduced traffic crash fatalities. But when public health advocates for saturation approaches do not acknowledge and measure disparities, these approaches may disproportionately burden under-resourced communities with the negative consequences of traffic stops. And, without some within-jurisdiction accountability, agencies are free to use their discretion to distribute DWI and seatbelt patrols into neighborhoods for other reasons. Those neighborhoods may not have the political and economic capital to fight in court, may not equitably weather the negative effects of such saturation interventions, and may not have the associated needs or see the consequent benefits to their public health outcomes.

### Limitations

This study has multiple limitations. Since only one agency enacted the intervention, our findings are suggestive but limited by sample size in many ways. For instance, in Fig. 2, because placebo tests are limited to the control pool of 8 non-intervention agencies, permutation *p*-values could only be in multiples of 0.125. Moreover, the relatively small control pool was only selected to provide adequate comparison to Fayetteville, i.e. by ensuring a spread of most metric around Fayetteville. Therefore, in some cases, some placebo trends and related tests were unstable for some metrics when no linear combination of other control agencies could remotely match the placebo agency. As example, no linear combination (weights adding to 100%) of smaller agencies can effectively model Charlotte, the largest agency with twice the population, twice the traffic stops, and three times the index crime count; if Charlotte were the agency of interest, it would require a different control pool.

Even in the case of Fayetteville, though the control pool provided adequate coverage for most metrics, one metric (the percent of Black non-Hispanic traffic stops) was best represented by a 100% weighted match against a single city agency in Durham, North Carolina. This effectively reduces the more nuanced synthetic control method to a simpler difference-in-difference model comparing a single intervention city to a single control city. In this case, Durham may be well suited as a control city to Fayetteville on most metrics (see Table 2) in this case, including closely matching this metric (e.g. 57% of traffic stop drivers are black in both cities in the pre-intervention period). However, this single control city analysis is not as robust to city-specific variation. If a group of agencies were to adopt this prioritization formally or smaller variations in these metrics were considered in a national study, results may be more robust. If a group of agencies were to adopt this prioritization formally or smaller variations in these metrics were considered in a national study, results may be more robust.

We do hypothesize that the synthetic control method improved confounding control compared to a simpler difference-in-difference model. However, an approach that incorporated data on more agencies and more covariates under a more detailed confounding control scheme would likely produce more accurate results than our approach of matching on the pre-intervention period. In this case, because of both small numbers of units and a lack of clarity on whether potential covariates were mediators or confounders of the intervention effect on each specific measure, we did not additionally adjust for metric-specific known confounders beyond the confounding control that metric-specific matching on the pre-intervention period provides. For example, while local economic changes associated with changes in a given metric (say, crime) across multiple cities would be adjusted for by comparison to the synthetic control built from cities matched on that crime metric, if Fayetteville had city-specific economic changes unrelated to those otherwise matched cities this analysis would not detect it. However, including time-unvarying or time-varying covariates requires the synthetic control to attempt to match both pre-intervention trends and covariates simultaneously; in sparse models with small sample sizes, this effectively deprioritizes unknown confounder control for (supposedly) known confounder control, should those covariates truly be confounders (and not mediators, etc.). While we did not have that causal clarity on covariates (or sample size) here to make that trade-off, other synthetic control studies with sufficient sample size and covariate clarity should include carefully chosen covariates to better control for local confounding otherwise uncontrolled for by pre-intervention matching. That said, particularly when there is a scarcity of implementation sites and promising interventions, documentation of aspiring anti-racist interventions is worthwhile in the face of these limitations (Jones et al. 2019).

Further, the capture of race-ethnicity in administrative datasets has known limitations (Knox and Lowe 2019). Race-ethnicity is a powerful social construct associated with many associated health disparities (Tsai and Venkataramani 2016), so many we that require dedicated frameworks to harmonize them (Duran and Pérez-Stable 2019). Because of its social construction (Ford and Airhihenbuwa 2018), the meaning of race-ethnicity changes over place and time and can vary person to person even within the same time and place. Health research acknowledges that self-identification may differ from social-identification (Jones et al. 2008). Even in the same person, conceptions of race-ethnicity change over the life course (Mihoko Doyle and Kao 2007). Concretely in this study, the self-identification options in justice databases are limited and may not match driver’s self-identity. Stopping officers may not refer to driver-specified race-ethnicity, notably incomplete in NC driver’s license records (Richard Stradling 2018), but instead fill out form SBI-122 based on their own ascription of the race of the driver. Indeed, there is documentation that in some regions law enforcement officers may knowingly misidentify race-ethnicity in response to scrutiny under new racial profiling laws and accountability that databases would seek to provide (Friberg et al. 2015).

## Conclusions

Reprioritizing traffic stops for public health can reduce negative crash outcomes, reduce disparities, and may not have negative impacts on crime. More generally, a public health anti-racist approach requires, for example and at least, that injury prevention researchers who design interventions that will be enacted by law enforcement (e.g., seatbelt traffic stop campaigns) to consider the reality that some agencies and officers may intentionally or unintentionally target populations in racially disparate ways. The collateral damage of even well-intentioned public safety interventions may outweigh their benefits. These damages may be disparately born by low-income and communities of color. Public safety and public health are intimately related endeavors, as evidenced by their relationship to traffic stops. When engaged with public safety issues, public health should adopt a critical view of policing at the same time both fields must critically interrogate their own historical and present-day practices. Conventional logics, such as the Ferguson Effect belief that de-prioritizing investigatory stops is associated with increases in violent crime, may not hold up to critical scrutiny.

Public health has outlined an explicit call to anti-racist practice and principles. Law enforcement organizations, individual law enforcement agencies and officers, city councils, county boards, and community groups may elect to take up that call to guide their own activities. When co-designing traffic stop programs, these groups should consider goals of equity and maximizing public health impact alongside effects on community trust. But regardless of law enforcement agency action or non-action, public health advocates can use traffic stop datasets to both ensure their efficacy for public safety goals and document and act on any racially disparate impacts of these programs.

## Supplementary information

**Additional file 1: Table S1.** Synthetic control weight vectors for each measure.

**Additional file 2: Table S2.** NC representativeness, access, and volume by race-ethnicity.

## Data Availability

The data that support the findings of this study are available from either publicly (e.g. US Census for demographic data, UNC HSRC for motor vehicle crash data at http://nccrashdata.hsrc.unc.edu/) or on request (e.g. NC SBI for crime or traffic stop data). Restrictions for datasets available on request, which were used under license for the current study, may apply, and so are available only through their requesting channels.

## Abbreviations

ACS: American Communities Survey
APHA: American Public Health Association
CARS: Crash Analysis Reduction Strategy
CDC: Center for Disease Control
COPS: Community Oriented Policing Service
CRI-TA: Collaborative Reform Initiative for Technical Assistance
CRT: Critical Race Theory
DDACTS: Data-Driven Approaches to Crime and Traffic Safety
DiD: Difference-in-difference
DWI: Driving while intoxicated
GPS: Global Positioning System
HSRC: Highway Safety Research Center
NHTS: National Household Travel Survey
NHTSA: National Highway Traffic Safety Administration
PHCRP: Public Health Critical Race Praxis
SBI: State Bureau of Investigation
TSRR: Traffic stop rate ratio
US: United States
VMT: Vehicle Miles Traveled
