# Supplementary material for: Re-prioritizing traffic stops to reduce motor vehicle crash outcomes and racial disparities
Source: Inj Epidemiol. 2020 Jan 20;7:3. doi: 10.1186/s40621-019-0227-6 (PMC6970293; doi:10.1186/s40621-019-0227-6)
Supplement: Supplementary file 1 — Additional file 1: Table S1. Synthetic control weight vectors for each measure. [file 40621_2019_227_MOESM1_ESM.docx]

Table S1 Synthetic control weight vectors for each measure.

Synthetic controls were programmatically determined by maximizing the match on pre-intervention trends for each measure, producing weight vectors of between one and five (mean 3.0) other NC city police departments linearly combined to model post-intervention counterfactual trends.

|  |  | **Cary** | **Charlotte** | **Durham** | **Greensboro** | **High Point** | **Raleigh** | **Wilmington** | **Winston-Salem** |
| --- | --- | --- | --- | --- | --- | --- | --- | --- | --- |
| **Traffic Stop Profile** | |  |  |  |  |  |  |  |  |
|  | Total Safety Stops | - | 4 | 75 | - | - | 21 | - | - |
|  | % Safety Stops | - | 7 | - | - | - | 93 | - | - |
|  | % Regulatory & Equip. Stops | - | 17 | - | - | - | 65 | - | 18 |
|  | % Discretionary | 31 | 58 | - | 7 | 4 | - | - | - |
|  |  |  |  |  |  |  |  |  |  |
| **Measures of Traffic Stop Disparity** | |  |  |  |  |  |  |  |  |
|  | % Black non-Hispanic Stops | - | - | 100 | - | - | - | - | - |
|  | Black non-Hispanic TSRR | 2 | 59 | 12 | - | 0 | - | 27 | - |
|  |  |  |  |  |  |  |  |  |  |
| **Motor Vehicle Crash Outcomes** | |  |  |  |  |  |  |  |  |
|  | Crashes (all) | 40 | - | - | - | - | 13 | - | 46 |
|  | Crashes (w/ injuries) | 34 | - | - | - | - | 31 | - | 35 |
|  | Traffic Fatalities | 26 | 31 | - | - | 43 | - | - | - |
|  |  |  |  |  |  |  |  |  |  |
| **Crime Outcomes** | |  |  |  |  |  |  |  |  |
|  | Violent Crimes | 29 | - | - | - | - | 3 | - | 67 |
|  | Violent Crime Rate (/1,000) | 14 | 49 | - | - | - | 11 | - | 26 |
|  | Index Crimes | 14 | - | - | - | - | - | - | 86 |
|  | Index Crime Rate (/1,000) | - | 15 | - | - | - | - | 17 | 68 |
