# Supplementary material for: Re-prioritizing traffic stops to reduce motor vehicle crash outcomes and racial disparities
Source: Inj Epidemiol. 2020 Jan 20;7:3. doi: 10.1186/s40621-019-0227-6 (PMC6970293; doi:10.1186/s40621-019-0227-6)
Supplement: Supplementary file 2 — Additional file 2: Table S2. NC representativeness, access, and volume by race-ethnicity. [file 40621_2019_227_MOESM2_ESM.docx]

Table S2 NC representativeness, access, and volume by race-ethnicity.

Data for North Carolina from 2017 National Household Travel Survey (NHTS). Black households have less access to vehicles, drive less often, and drive fewer total vehicle miles than White non-Hispanic drivers. Measures marked with a * were used in adjusting residential counts to approximate vehicle miles traveled for rate calculations. Reprinted from Fliss, 2019.

|  | **Measures of Survey Representation** | | |
| --- | --- | --- | --- |
| **Race-Ethnicity** | **Number surveyed** | **Number represented** | **Number drivers represented** |
| Asian | 307 | 251,577 | 184,748 |
| American Indian / Alaskan Native | 156 | 78,171 | 57,496 |
| Black / African American | 2,444 | 2,015,261 | 1,294,804 |
| Hispanic | 600 | 828,660 | 532,834 |
| Other | 522 | 324,620 | 199,508 |
| White non-Hispanic | 13,556 | 5,950,650 | 4,894,298 |
| **Total** | 17,585 | 9,448,939 | 7,163,689 |
|  |  |  |  |
|  |  |  |  |
|  | **Measures of Access** | | |
| **Race-Ethnicity** | **Household has personal vehicle access (%)** | **Household vehicle use at least a few times a month (%)** | **Any driving during year (%)** |
| Asian | 99.8 | 99.0 | 73.4 |
| American Indian / Alaskan Native | 90.3 | 95.4 | 73.6 |
| Black / African American | 85.3 | 88.2 | 64.2 |
| Hispanic | 97.0 | 97.2 | 64.3 |
| Other | 96.1 | 97.6 | 61.5 |
| White non-Hispanic | 98.4 | 98.0 | 82.2 |
| **Total** | 95.8 | 96.2 | 76.8 |
|  |  |  |  |
|  |  |  |  |
|  | **Measures of Driver VMT** | | |
| **Race-Ethnicity** | **Annual VMT per driver (miles)** | **Annual VMT per person (miles)** | **Average miles per trip (miles)** |
| Asian | 8,677 | 6,372 | 10.0 |
| American Indian / Alaskan Native | 12,219 | 8,987 | 10.8 |
| Black / African American | 9,775 | 6,280 | 9.7 |
| Hispanic | 12,434 | 7,995 | 12.4 |
| Other | 8,762 | 5,385 | 8.6 |
| White non-Hispanic | 10,819 | 8,898 | 10.4 |
| **Total** | 10,649 | 8,196 | 10.4 |
